# Modelling host- *Trypanosoma brucei gambiense* interactions *in vitro* using human induced pluripotent stem cell-derived cortical brain organoids

**DOI:** 10.12688/f1000research.131507.2

**Published:** 2023-07-28

**Authors:** Praveena Chandrasegaran, Agatha Nabilla Lestari, Matthew C. Sinton, Jay Gopalakrishnan, Juan F. Quintana

**Affiliations:** 1 School of Biodiversity, One Health, and Veterinary Medicine (SBOHVM), University of Glasgow, Glasgow, Scotland, G61 1QH, UK; 2 Wellcome Centre for Integrative Parasitology (WCIP), University of Glasgow, Glasgow, Scotland, G61 1QH, UK; 3 Laboratory of Centrosome and Cytoskeleton Biology, Laboratory for Centrosome and Cytoskeleton Biology, Institute für Humangenetik, Universitätsklinikum Düsseldorf, Heinrich-Heine-Universität, Düsseldorf, 40225, Germany

**Keywords:** Brain organoids, sleeping sickness African trypanosomes, brain infection, in vitro culture

## Abstract

**Background:** Sleeping sickness is caused by the extracellular parasite *Trypanosoma brucei* and is associated with neuroinflammation and neuropsychiatric disorders, including disruption of sleep/wake patterns, and is now recognised as a circadian disorder. Sleeping sickness is traditionally studied using murine models of infection due to the lack of alternative *in vitro* systems that fully recapitulate the cellular diversity and functionality of the human brain. The aim of this study is to develop a much-needed *in vitro* system that reduces and replaces live animals for the study of infections in the central nervous system, using sleeping sickness as a model infection.

**Methods: **We developed a co-culture system using induced pluripotent stem cell (iPSC)-derived cortical human brain organoids and the human pathogen *T. b. gambiense* to model host-pathogen interactions *in vitro*. Upon co-culture, we analysed the transcriptional responses of the brain organoids to *T. b. gambiense* over two time points.

**Results: **We detected broad transcriptional changes in brain organoids exposed to *T. b. gambiense*, mainly associated with innate immune responses, chemotaxis, and blood vessel differentiation compared to untreated organoids.

**Conclusions: **Our co-culture system provides novel, more ethical avenues to study host-pathogen interactions in the brain as alternative models to experimental infections in mice. Although our data support the use of brain organoids to model host-pathogen interactions during *T. brucei* infection as an alternative to *in vivo* models, future work is required to increase the complexity of the organoids ( *e.g.*, addition of microglia and vasculature). We envision that the adoption of organoid systems is beneficial to researchers studying mechanisms of brain infection by protozoan parasites. Furthermore, organoid systems have the potential to be used to study other parasites that affect the brain significantly reducing the number of animals undergoing moderate and/or severe protocols associated with the study of neuroinflammation and brain infections.

Research highlights **Scientific benefit(s)**

• Human-derived brain organoids can be used to study neuropathogenesis during *Trypanosoma* infection. This has been challenging to study in human tissues due to ethical implications and lack of complex *in vitro* culture systems.
• Evaluation of putative human brain cell populations associated with innate responses to protozoan pathogens.

**3Rs benefit(s)**

• Adoption of stem cell-derived 3D organoids can reduce ~47% of the mice used to study trypanosome infection, which would typically undergo protocols considered moderate or severe.
• Further ~20% reduction of donor mice required to generate infectious parasites, which is a moderate procedures.

**Practical benefit(s)**

• Possible to effectively introduce mutations of interest into the organoids without the need to established complex and expensive breeding schemes.
• Reductions in the number of animals required for *in vivo* work reduces breeding and husbandry costs.

**Current applications**

• Evaluation of global responses to a human pathogen.

**Potential applications**

• Screening for drugs acting on the CNS for treatment of infectious diseases.
• Can be combined with additional organoids, cell type/s of interest (“building blocks”), and/or organic matrices or scaffolds to generate more complex tissues/organs.
• Potential to manipulate genes/pathways ( *e.g.*, CRISPR-Cas9 gene editing) to assess their function in pathogenesis to infection.

## Introduction

Neurotropic pathogens encompass a wide range of parasitic organisms, from viruses to protozoan parasites, and are the causative agents of debilitating conditions affecting the central nervous system (CNS), often resulting in life-long impairments and death if left untreated. To date, most of these infections are studied using murine models of infection. Although these infection models often recapitulate the clinical outcomes observed in humans, there are serious ethical and biological implications associated with *in vivo* host-pathogen interaction studies. More recently, the generation of organoids developed *in vitro* from human stem cells have provided novel insights into developmental biology, and their potential application as alternative models to study host-pathogen interactions is starting to be recognised. These models offer an opportunity to interrogate human tissues that are difficult to access, such as CNS tissue. Indeed, human brain organoids comprising the diversity of cell types representative of the complex neuroepithelium are an increasingly attractive model system to interrogate how human nervous cells respond to infection. Currently, *in vitro* brain organoid systems are being used to study infections from ZIKA and SARS-CoV-2, ^1^^–^^4^ and have proven insightful for understanding other parasitic infections, including toxoplasmosis and malaria. ^5^^,^^6^ However, these *in vitro* systems have not been used to explore the pathogenesis of human African trypanosomiasis, a parasitic infection traditionally known for its devastating neurological effects. ^7^^–^^10^

Here, we explored whether human brain organoids can be used to model host-trypanosome interactions *in vitro.* Using bulk RNA sequencing, we observed that the human cortical brain organoids transcriptionally respond to the human pathogen *T. brucei gambiense* by upregulating gene pathways associated with innate immune functions, amongst others. Some of the upregulated genes are proposed to have antimicrobial properties, suggesting that human brain organoids are able to sense and respond to pathogens in the absence of innate immune cells ( *e.g.*, microglia). Using this novel *in vitro* system, we estimate a direct reduction of ~47% of animals required to achieve similar conclusions, and ~20% of animals used as donors to generate infectious parasites. The methods and results presented here have the potential to open new research avenues for the adoption of human brain organoids to model host-pathogen interactions with important implications for the 3Rs principles—replacement, refinement, and reduction.

## Methods

### Cerebral organoid generation and *in vitro* culture

This work was conducted jointly at the Heinrich-Heine-Universität and the University of Glasgow.

1. IMR90 human induced pluripotent embryonic stem cells ( IPS(IMR90)-2 (RRID:CVCL_C435)), maintained at 80% confluency, were seeded at ~10,000 cells per well in mTeSR1 (Stem Cell Technologies, Vancouver, Canada) in 24-well, Matrigel-precoated plates (Corning, NY, USA). Cells were incubated at 37°C and 5% CO _2_ with medium changed daily.
2. Colonies were observed to form after 7-10 days.
3. To detach colonies, mTeSR1 media was removed and the wells were washed once with 1 ml of 1X D-PBS without Calcium and Magnesium (Stem Cell Technologies) at 37°C.
4. PBS was removed and discarded. A total of 1 ml ReLeSR™ at 37°C temp (Stem Cell Technologies) was added per well.
5. Plates were incubated for 5-7 minutes at 37°C, after which 1ml TeSR™ was added to each well and the plates were vortexed for 2-3 minutes at room temperature (17-22°C) until the cultures were fully detached. Note that the mean aggregate size should be approximately 50-200 μm.
6. Cell pellets were resuspended in 500 μl AggreWell medium and 10% Clone R (Stem Cell Technologies) and seeded at 9,000 cells per well in round-bottom 96-well plates (in ~200 ml) and incubated at 37°C and 5% CO _2_.
7. After two days, the medium was replaced with fresh AggreWell without Clone R, and the cells were incubated for an additional three days at 37°C and 5% CO _2_. The medium was changed by gently placing the plate in a 45° angle to medium change.
8. On day six, the medium was replaced with neural induction medium (NIM) containing DMEM/F12, N2 supplement (Thermo Fisher Scientific, Waltham, MA, USA), minimum essential medium-nonessential amino acids (MEM-NEAAs), GlutaMAX (Thermo Fisher Scientific) and 1 μg/ml heparin (Sigma, MO, USA), and the cells incubated for five days 37°C and 5% CO _2_.
9. After the NIM medium was changed, embryoid bodies (EBs) were transferred by pipette onto Matrigel droplets (Corning) that were 3 mm in diameter on the inner part of a 75 mm petri dish. The EBs should appear as located at the centre of the droplet. These droplets were incubated for 1 hour at 37°C, transferred to 24-well plates and maintained in cerebral organoid differentiation medium (CORD) containing DMEM/F12 and Neural Basal Medium (in 1:1 ratio), supplemented with 1:200 N2 (Thermo Scientific), 1:100 l-glutamine (Stem Cell Technologies), 1:100 B27 without vitamin A (Thermo Scientific), 100 U/ml penicillin, 100 μg/ml streptomycin, 23 μM insulin (Sigma-Aldrich), 0.05 mM MEM non-essential amino acids (NAA), and 0.05 mM β-mercaptoethanol (Life Technologies) was used to differentiate the Matrigel embedded droplets. The medium was replaced every three days until usage (∼2 month in culture to ensure organoid maturation).

### *Trypanosoma brucei gambiense in vitro* culture

1. Culture-adapted bloodstream slender forms of *Trypanosoma brucei gambiense* Eliane strain (MHOM/CI/52/ITMAP 2188) ^11^ were used in all experiments. This strain, originally isolated from an infected patient in Côte d’Ivoire (Ivory Coast) ^11^ was previously adapted in the laboratory to grow in HMI-9 culture medium supplemented with 20% foetal calf serum (FCS).
2. Cultures were maintained at 37°C in humidified atmosphere containing 5% CO _2_. Pleomorphic parasites were typically maintained at a density of 10 ^5^ and 10 ^6^ parasites/ml at 37°C and 5% CO _2_.

### *Trypanosoma brucei gambiense* - human brain organoids co-culture system

**Figure 1.**
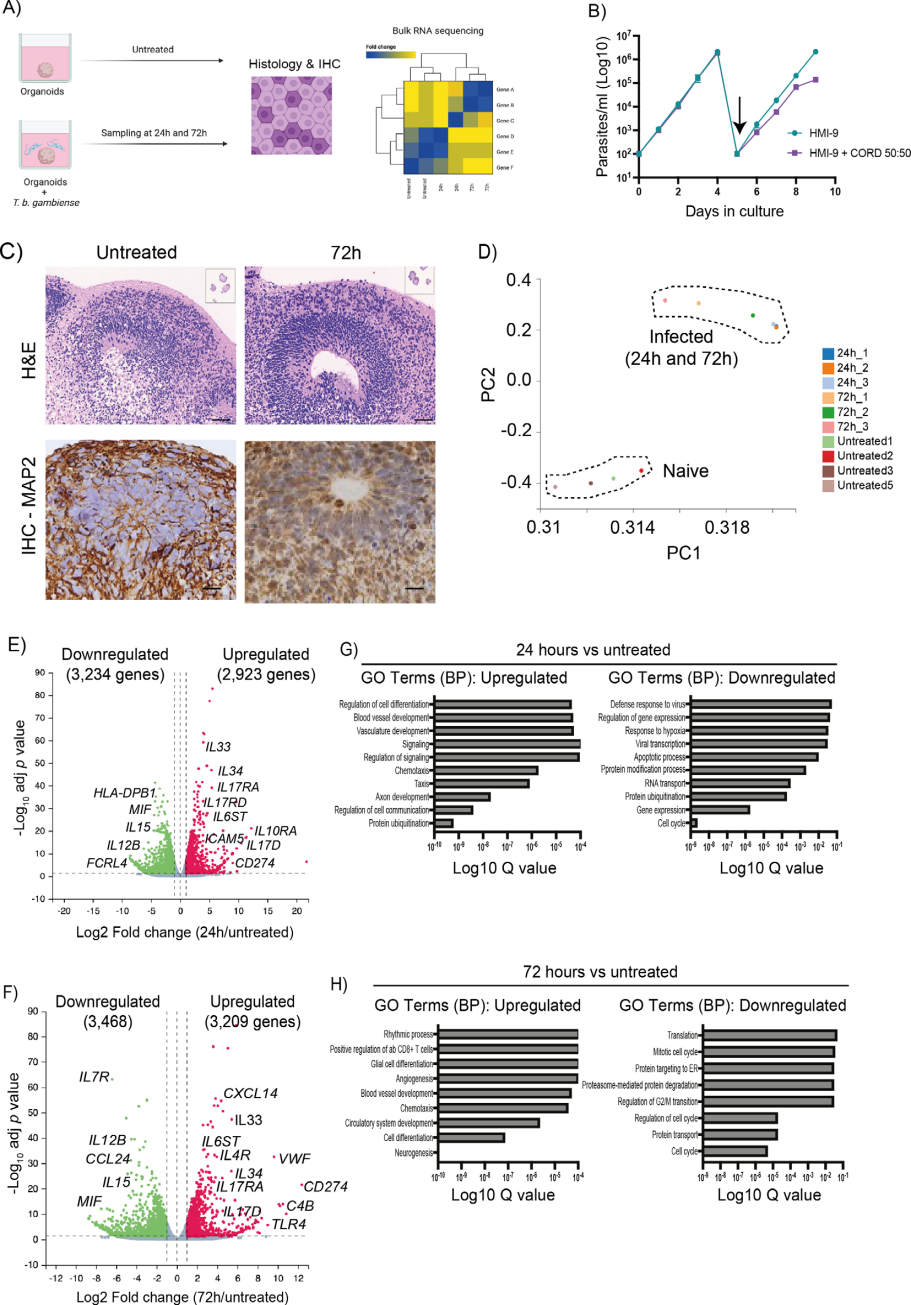
Bulk transcriptomics analysis of human cortical brain organoids co-cultured with *T. brucei gambiense.* A) Schematic representation of the experimental design developed for this study. B) Growth curve assay for *T. b. gambiense* in HMI-9 alone (teal) or diluted 50:50 with CORD media (magenta). The arrow indicates a dilution step to bring the parasite cultures down to 10 ^2^ parasites/ml. Data shown as mean ± standard deviation from three independent experiments. C) Representative H&E staining and MAP 2 Immunohistochemistry from naïve (left) and *T. brucei gambiense*-infected (right) organoid after 72 hours of *in vitro* co-culture. Scale bar = 50 μm. D) Principal component analysis of the samples including the bulk RNA sequencing analysis. Volcano plot of differentially expressed genes between untreated organoids and after (E) 24 hours and (F) 72 hours in culture with T *. brucei gambiense.* Dotted line represents the significance (-0.5 < Log _2_FC > 0.5 and *p* adjusted value < 0.05). Pathway analysis of the genes dysregulated at (G) 24 hours and (H) 72 hours in culture with *T. brucei gambiense.* The adjusted *p* value (Q value) for each of the enriched pathways is included. MAP 2, microtubule associated protein 2.

1. A total of 10 ^5^ parasites at log-phase of growth were co-cultured with the brain organoids on 12 well plates (Final ratio of 1 organoid:10 ^5^ parasites per well) for a period of 24 or 72 hours in HMI-9 media diluted 50:50 with CORD media at 37°C and 5% CO _2_ in round-bottomed 96-well plates. These two time points were chosen to mimic acute (24 hours) and chronic (72 hours) responses and we determined that parasites grew well under these conditions, at least during the first 72 hours in culture ( Figure 1B).
2. In parallel, organoids cultured in HMI-9 media diluted 50:50 with CORD media at 37°C and 5% CO _2_ but without parasites were also seeded in round-bottomed 96-well plates and were included as controls to assess the effect of diluted media on the organoids transcriptome. As controls, we included organoids kept in 50:50 HMI-9:CORD organoid media alone.
3. After 24 or 72 hours, the organoids were gently washed 5 times with 1X PBS (Gibco) and wide bore pipette tips to remove parasites. Then, some organoids were fixed in 4% PFA for 24 hours at room temperature and preserved as paraffin-embedded blocks for immunohistochemistry analysis. The rest of the organoids were processed for bulk transcriptomics.

### Immunohistochemistry

1. Paraformaldehyde-fixed organoid were processed into paraffin blocks for long-term storage.
2. We prepared 5 μm thick paraffin sections, which were placed on Superfrost Plus™ slides (Fisher Scientific) and stained with Mayer’s haematoxylin Solution (Sigma-Aldrich), Bluing Buffer (Dako) for 5 minutes and 1:10 dilution of Eosin Y solution (Sigma-Aldrich) in 0.45 M of Tris-acetic acid buffer, pH 6.0, for 5 minutes in distilled water, with 3-4 washing steps in ultrapure water between each step. All solutions were kept at room temperature. The H&E staining was conducted using a Dako Autostainer Link 48 (Dako) with all the incubation steps at room temperature (17-22°C).
3. For staining with the monoclonal neuron-specific microtubule associated protein 2 (MAP 2, Clone M13, Thermo Fisher Scientific Cat. No. 13-1500. RRID: AB_2533001), 5 μm thick paraffin sections were treated in a pressure cooker (~140°C) for 5 minutes in citrate buffer pH 6.0, followed by staining with the monoclonal NSE antibody (Cell Signalling Technologies, clone E2H9X, Cat. No. 24330. RRID: AB_2868543) diluted in 1:1,000 in 1× blocking buffer (Dako) and incubated overnight at 4°C. Staining was conducted using a goat anti-Rabbit antibody coupled to Horseradish peroxidase (1:1,000, Thermo Fisher Scientific Cat. No. A16104. RRID: 2534776) for 1 hour at room temperature.
4. The samples were mounted in VECTASHIELD Antifade Mounting Media with DAPI (Vectorlabs, Cat. No. H-1200. RRID: AB_2336790) and visualised on a Axio Imager 2 instrument (Zeiss. RRID: SCR_018876).

### Bulk RNA sequencing and data analysis

1. Before proceeding with the RNA extraction step, all the pipettes and surfaces were thoroughly cleaned with RNAZap (Thermo) to remove RNAses. For this protocol, we used sterile filtered tips.
2. At the selected time points, brain organoids (n = 3 replicates / experimental condition) were harvested and washed in 500 μl of 1X PBS at 4°C twice. The washes were conducted by letting the organoids settle at the bottom of a 1.5 ml Eppendorf tube before removing the supernatant.
3. Once washed, the organoids were resuspended in 500 μl of Qiazol (Qiagen) and dissociated firmly by pipetting up and down using a wide bore p1000 pipette tip.
4. The dissociated tissue was then subjected to total RNA extraction using the mRNeasy kit (Qiagen), following the recommended volume of chloroform. All the solutions were kept at room temperature (17-22°C) unless indicated otherwise by the manufacturer. All the centrifugation steps were conducted at 4°C. We eluted the total RNA from brain organoids in 50 ml of EB buffer (Qiagen). On average, we detected a recovery of ~100 μg/ml of total RNA, as quantified by Qubit.
5. The quality of the RNA was assessed on a Bioanalyzer RNA Pico chip (Agilent). We considered an RNA Integrity Number (RIN) value >8.0 to be ideal for bulk RNA sequencing. All the samples analysed here consistently had a RIN value >8.0.
6. Once assessed, 1 μg of total RNA per sample was submitted to the Beijing Genomic Institute (BGI; RRID: SCR_011114) for RNA sequencing and processed for 150 bp paired-end sequencing on the DNBSeq platform (RRID: SCR_017981).
7. Once sequenced, raw reads were filtered using SOAPnuke (RRID:SCR_015025) software (v1.5.2) developed by BGI, allowing for the removal of reads containing adapters, reads with N content >5%, or with a base quality score <15.
8. Clean reads were then aligned the to the human genome using the package Hierarchical Indexing for Spliced Alignment of Transcripts (HISAT) (RRID:SCR_015530) (v2.0.4) and Bowtie 2 (RRID:SCR_016368) (v2.2.0), with default parameters using the Genome reference consortium Human Build 38 patch release 12 (GRCh38.p12).
9. Subsequent downstream analysis, including differential gene expression and pathway analysis using Gene Ontology (RRID: SCR_002811), were conducted on the Dr. Tom analysis suite built by BGI. For gene expression analysis, differentially expressed genes were considered significant if the adjusted *p* value < 0.05, and Log _2_ fold change of -2< or >2.

## Results

African trypanosomes cause extensive neurological changes resulting in neuropsychiatric disorders and culminating in death if not treated adequately. Although this disease is frequently modelled using experimental infections in mice, for ethical reasons around the use of animals in research, we were motivated to explore the utility of induced pluripotent stem cell (iPSC)-derived human cortical brain organoids to model brain-trypanosome interactions *in vitro* as alternatives to *in vivo* infections. Thus, using an *in vitro* co-culture system, we set out to characterise the transcriptional responses of the iPSC-derived human brain organoids to the human pathogen *T. brucei gambiense* ( Figure 1A), ^22^^,^^23^ compared to organoids that were not incubated with the parasites. These time points were selected to evaluate early (24 hours) and late (72 hours) responses, in an attempt to gain as much insight as possible into the temporal dynamics associated with tissue responses to infection. Importantly, we did not detect significant morphological or histological changes in the organoids exposed to the parasites based on the morphological aspects observed upon H&E staining and MAP2 staining ( Figure 1C), suggesting that *T. brucei gambiense* does not seem to elicit tissue damage over a 72 hour culture period, at least not with the markers used in this study. Principal component analysis demonstrates that at a transcriptional level, the samples segregate mainly based on infection status and experimental time point, but with limited transcriptional variation between samples harvested at 24 and 72 hours ( Figure 1D). In these brain cortical organoids, we identified a total of 6,157 dysregulated genes at 24 hours (3,234 and 2,923 downregulated and upregulated genes, respectively) and 6,677 dysregulated genes at 72 hours (3,468 and 3,209 downregulated and upregulated genes, respectively) ( Figure 1E and 1F, and Table S1 in *Underlying data*), ^22^^,^^23^ defined as genes with an adjusted *p* value < 0.05 and a Log _2_ Fold change of -2< or >2. To obtain a broad overview of immune related pathways, we examined cytokine, chemokine, and immune receptors that were significantly dysregulated in brain organoids exposed to *T. b. gambiense.* We detected several genes with canonical immune functions such as *CD274*, that encodes for Programme death ligand 1(PD-L1), the complement factor *C4B*, and the glial fibrillary acidic protein ( *GFAP)*, typically associated with gliosis during CNS inflammation ^12^ ( Table 1 and Table S2 in *Underlying data*). ^22^^,^^23^ Additionally, we detected the expression of several interleukins and chemokines such as interleukin-34 ( *IL34)* that promotes monocyte and macrophage survival and differentiation, ^13^ the chemokine *CXCL14* involved in immune cell recruitment, ^14^ transforming growth factor beta 1 ( *TGFB1*), and the alarmin *IL33*, which is a critical mediator of innate immune responses and inflammation ^15^ ( Table 1 and Table S2 in *Underlying data*). ^22^^,^^23^ We also detected significant expression of the interleukin-17 receptor subunit A and D ( *IL17RA* and *IL17RD*, respectively), interleukin-10 receptor subunit a ( *IL10RA*), and the Interferon gamma receptor 1 ( *IFNGR1*) ( Table 1 and Table S2 in *Underlying data*), ^22^^,^^23^ indicating that these organoids are primed to sense and respond to *T. brucei gambiense* by activating IL-17, IL-10, and IFNγ signalling pathways. Furthermore, we also detected genes involved in angiogenesis and endothelial function, including the vascular endothelium growth factor subunit c ( *VEGFC*), cadherin 5 ( *CDH5*), the integrin associated protein *CD47*, and von Willebrand factor ( *VWF*) ( Table 1 and Table S2 in *Underlying data*), ^22^^,^^23^ suggesting that co-culture with *T. b. gambiense* also induces the expression of genes associated with vasculogenesis and vascular repair. Some of these genes showed a temporal expression dynamic, with some genes involved in immune sensing, recruitment and tissue repair ( *e.g.*, *CXCL14*, *VWF*, *TLR4*, *IL4R*) being exclusively detected after 72 hours of exposure to *T. b. gambiense* compared to naïve controls.

**Table 1.** Upregulated immune-related genes in iPSC-derived human brain organoids in response to *Trypanosoma brucei gambiense.*

Gene of interest	24 h co-culture		72 h co-culture	
	Log2FC	Adjusted *p* value	Log2FC	Adjusted *p* value
*CD274*	6.316	1.33 ^-10^	5.578	1.26 ^-07^
*C4B*	5.734	2.29 ^-03^	8.151	1.86 ^-08^
*IL34*	3.909	1.73 ^-10^	3.522	4.41 ^-08^
*IL33*	1.241	8.75 ^-04^	1.389	5.55 ^-04^
*IL17D*	0.8711	0.0351	ND	ND
*IL6ST*	0.694	0.0270	1.070	9.66 ^-04^
*CXCL14*	ND	ND	2.123	2.74 ^-04^
*VWF*	ND	ND	9.765	6.60 ^-03^
*VEGFC*	1.480	0.01404	1.886	3.55 ^-04^
*CCL25*	2.723	0.0453	ND	ND
*IFITM1*	2.277	7.10 ^-03^	3.168	9.28 ^-16^
*IL10RA*	1.585	7.93 ^-03^	ND	ND
*IL17RD*	1.512	1.29 ^-07^	1.582	1.24 ^-08^
*IL17RA*	1.191	3.27 ^-03^	1.384	6.34 ^-04^
*IL1RL1*	ND	ND	5.857	0.016
*IL4R*	ND	ND	3.109	0.0452
*IL13RA1*	1.887	3.90 ^-03^	1.986	9.32 ^-04^
*TLR4*	ND	ND	5.05	0.0315
*GFAP*	3.642	1.7- ^09^	3.363	7.96 ^-09^
*TGFB1*	0.9907	8.82 ^-03^	1.22	6.99 ^-03^
*IFNGR1*	ND	ND	0.605	0.043
*CD47*	1.225	2.41 ^-06^	1.151	2.80 ^-05^
*CDH5*	ND	ND	7.178	0.015

iPSC = induced pluripotent stem cell; ND = Not detected; h = hours.

To gain a better understanding of the broad transcriptional responses triggered in the human brain organoids to *T. b. gambiense infection*, we performed Gene Ontology analysis on genes significantly dysregulated. After 24 hours of exposure to *T. b. gambiense*, the iPSC-derived human brain organoids upregulate genes associated with blood vessel and vasculature development, signalling, and chemotaxis, with a concomitant reduction in genes associated with response to hypoxia, defence response against viruses, and protein ubiquitination ( Figure 1G). At 72 hours, the pathways overrepresented in the organoids transcriptome were associated with glial cell differentiation, positive regulation of CD8 ^+^ T cells, chemotaxis, and vascular and blood vessel differentiation, and a significant reduction of gene pathways associated with cell cycle progression, protein transport and proteasome-mediated protein degradation ( Figure 1H). Taken together, these data suggest that *T. b. gambiense* trigger a broad innate-like immune response in the iPSC-derived human brain organoids accompanied by upregulation of genes involved in vascular development, immune chemotaxis, and cytokine-mediated immune signalling.

### Use cases

Similar approaches have been recently implemented to study host-pathogen interactions in the context of viral infections and protozoan infections, including toxoplasmosis and malaria, *in vitro.* We anticipate that our detailed protocol can be used to explore further interactions between *T. brucei* and iPSC-derived human brain organoids in more detail, including novel effects of *T. brucei* on the function of human neurons, which remains unexplored. We additionally anticipate that the protocol provided here can be leveraged to study potential cytotoxic side effects of novel antiparasitic compounds.

## Discussion and outlook

In this study, we tested the possibility of using stem cell-derived human brain organoids as an *in vitro* system as an alternative mode to live animals to study host-trypanosome interactions, in line with the 3Rs principles. We firstly set up an *in vitro* co-culture system whereby iPSC-derived human brain organoids were co-cultured with the human pathogen *T. b. gambiense* and assessed the response of these organoids to the pathogen using histology and transcriptomics as a proxy for global responses to the pathogen. The data presented here demonstrate that iPSC-derived human brain organoids trigger a transcriptional programme associated with an innate-like immune response when exposed to *T. brucei gambiense.* Bulk transcriptomics has enabled us to identify that the brain organoids specifically respond to *T. brucei gambiense in vitro* by upregulating several genes with putative immune functions such as *CXCL14*, the alarmin *IL33*, the complement component *C4B*, as well as vasculogenesis and vascular repair such as *VEGFC*. *CXCL14* is a potent antimicrobial cytokine secreted in response to inflammatory processes and is critical for human neutrophil recruitment, ^14^^,^^16^^,^^17^ which have been proposed as important players in controlling CNS infections. ^18^^,^^19^ Similarly, the upregulation of several genes critical for angiogenesis and development of vascular beds, including *VEGFA, VEGFB*, and *VWF*, suggests that they may potentially support vasculogenesis in the presence of this pathogen. All of these observations require further testing at the protein and functional level but provide an initial robust framework to dissect the relevance of these 3D culture systems to model brain-trypanosome interactions.

Our work provides an initial approach to explore the utility of complex 3D culture systems to study host-Trypanosoma interactions *in vitro,* adding African trypanosomes to the compendium of pathogens that have been tested to model host-pathogen interactions using brain organoids. However, there are many challenges and considerations that need to be addressed for the full implementation of these *in vitro* systems, with the aim of replacing animal models of infection. One of the critical hurdles is to generate fully mature organs *in vitro*, encompassing all the cell types typically identified *in vivo*, including microglia and vasculature cells, ^20^ which are likely to be the main drivers of and/or responders to infection. The incorporation of additional organoids ( *e.g.*, vascular or choroid plexus organoids ^1^) or inclusion of additional cell types ( *e.g.*, endothelial cells, microglia), referred to as “building blocks”, ^21^ will support the development of mature cortical brain organoids that could faithfully recapitulate the immunological responses observed *in vivo.* Given these technical and biological limitations, we are unable to examine the role of these cell types using our *in vitro* host-pathogen culture system. Future work addressing these key challenges will improve the quality of these organoids to model CNS infections *in vitro*, facilitating the reduction and/or replacement of animals in research. Our work provides a wealth of data that can be further mined to design, refine, or implement *in vitro* experiments ( *e.g.*, using stem-cell derived astrocytes) as alternatives for *in vivo* work, and provides a foundation for future work in this area.

In summary, we delivered an initial proof-of-concept framework for future adoption of these *in vitro* systems for neuro-immunology research, motivated by the need to reduce and/or fully replace to use animals to study brain-pathogen interactions, in line with the 3Rs principles under the Animals (Scientific procedure) Act, 1986. Based on our estimations, with this model in place, animals used to study brain infections with African trypanosomes, typically considered to be moderate to severe procedures, would have been reduced by ~47%, with an additional ~27% reduction in the number of immunocompromised mice used as donors to generate infectious parasites.

## Data Availability

Gene Expression Omnibus: Modelling host- *Trypanosoma brucei gambiense* interactions in vitro using human induced pluripotent stem cell-derived cortical brain organoids. Accession number GSE220766; https://identifiers.org/geo:GSE220766. ^22^ Figshare: Modelling host- *Trypanosoma brucei gambiense* interactions in vitro using human induced pluripotent stem cell-derived cortical brain organoids. https://doi.org/10.6084/m9.figshare.22491100. ^23^ This project contains the following underlying data:
‐
Table S1 (Quality control and summary of the bulk transcriptomics analysis obtained from the iPSC-derived human brain organoids co-culture with *T. b. gambiense*) ‐
Table S2 (List of differentially dysregulated genes in iPSC-derived human brain organoids at 24 h and 72 h in co-culture with *T. b. gambiens*) Data are available under the terms of the Creative Commons Attribution 4.0 International license (CC-BY 4.0).
