# Supplementary material for: Modelling host- Trypanosoma brucei gambiense interactions in vitro using human induced pluripotent stem cell-derived cortical brain organoids
Source: F1000Res. 2023 Jul 28;12:437. Originally published 2023 Apr 24. [Version 2] doi: 10.12688/f1000research.131507.2 (PMC10425695; doi:10.12688/f1000research.131507.2)
Supplement: Peer review history

# Peer review: Reviewer response for version 2

**DOI:** 10.5256/f1000research.153520.r191610

**Published:** 2023-08-14

**Authors:** Maria Bernabeu

**Affiliations:** 1 Tissue Biology and Disease Modelling Unit, European Molecular Biology Laboratory (EMBL), Barcelona, Spain

I endorse the indexing of the article but there is indeed a wrong statement on summary of changes: "We have removed *PECAM1* from the text and table as a marker for endothelial cells". PECAM1 is a marker for endothelial cells, EPCAM is not. EPCAM is a marker for epithelial cells. So I'm not sure why the authors deleted PECAM1 and kept EPCAM.

Is the rationale for developing the new method (or application) clearly explained?

Yes

Are the 3Rs implications of the work described accurately?

Yes

Is the description of the method technically sound?

Yes

Are the conclusions about the method and its performance adequately supported by the findings presented in the article?

Yes

If any results are presented, are all the source data underlying the results available to ensure full reproducibility?

Yes

Are a suitable application and appropriate end-users identified?

Yes

Are sufficient details provided to allow replication of the method development and its use by others?

Partly

Reviewer Expertise:

3D brain bioengineered models, host-parasite interactions, malaria, vascular engineering

I confirm that I have read this submission and believe that I have an appropriate level of expertise to confirm that it is of an acceptable scientific standard.

# Peer review: Reviewer response for version 2

**DOI:** 10.5256/f1000research.153520.r191612

**Published:** 2023-08-01

**Authors:** David Smith

**Affiliations:** 1 Moredun Research Institute, Penicuik, Scotland, UK

Thank you to the authors for addressing the comments raised in the initial review.

 I consider all comments raised to have been addressed, either by manuscript edits or direct responses to the reviewer report. I therefore confirm support for the indexing of this publication.

Is the rationale for developing the new method (or application) clearly explained?

Yes

Are the 3Rs implications of the work described accurately?

Yes

Is the description of the method technically sound?

Yes

Are the conclusions about the method and its performance adequately supported by the findings presented in the article?

Yes

If any results are presented, are all the source data underlying the results available to ensure full reproducibility?

Yes

Are a suitable application and appropriate end-users identified?

Yes

Are sufficient details provided to allow replication of the method development and its use by others?

Partly

Reviewer Expertise:

Organoids, parasitology, host:parasite interactions, protozoa, helminths

I confirm that I have read this submission and believe that I have an appropriate level of expertise to confirm that it is of an acceptable scientific standard.

# Peer review: Reviewer response for version 1

**DOI:** 10.5256/f1000research.144355.r170807

**Published:** 2023-05-18

**Authors:** Maria Bernabeu

**Affiliations:** 1 Tissue Biology and Disease Modelling Unit, European Molecular Biology Laboratory (EMBL), Barcelona, Spain

In this manuscript, Chandrasegaran *et al.*, use for the first-time *in vitro *brain organoids to understand *Trypanosoma brucei gambiense *pathogenesis. This is an important topic, as brain organoids have already become as a valid strategy to study disease mechanisms. Although the team led by Juan Quintana is already a leader in animal models of *T. brucei,* I would like to praise their efforts on using alternative disease modelling tools that could reduce the number of research animals and overcome differences between mouse and humans. The authors show differential gene expression after brain organoid incubation with *T, brucei gambiense *after a 24h and 72h incubation. The manuscript is timely, and overall, scientifically sound and well written. I support the article indexing after the introduction of some modifications on the text.

**Major**

- Although the methods section is very clear, I couldn’t find any reference on how long the brain organoids were grown. This is important because brain organoids are grown for long periods of time (up to a year) and depending on the maturation time, they acquire different brain developmental stages. I would suggest that the authors include this information in the methods section, as well as on the results or discussion section, as it is important for interpretation of the results.
- How does the 24h and 72h time point or the parasite concentration used compare with the life cycle of *T. brucei gambiense* in the brain? This information could be useful for an audience not familiar with *T. brucei*.
- The authors couldn’t find any brain damage markers on Fig 2C after staining with two different markers. However, other non-used markers could be affected. Although the sentence in the result section mildly implies that (" *Importantly, we did not detect significant morphological or histological changes in the organoids* *exposed to the parasites based on H&E staining and MAP 2 staining (Figure 1C), suggesting that T. brucei gambiense does not elicit tissue damage over a 72 hour culture period.*”), I would suggest to rephrase to make it more obvious. Furthermore, did the author quantify for differences in expression on MAP2, or is the analysis mostly qualitative?
- The increased expression of endothelial markers is intriguing, taking into account that brain organoids present minimal presence of endothelial cells. This is important because vascularization of brain organoids is one of the main challenges in the bioengineering field, and the presence of vasculature in these models could be used to study the molecular mechanisms of *T. brucei* crossing through the brain microvasculature. Did the authors checked for the presence of blood vessels by immunostaining. In a similar topic, I would like to clarify that EPCAM is not an endothelial marker, so I would recommend to delete any reference to EPCAM in the figures/tables, results and discussion section.

**Minor**

- Abstract *“Future work is required to increase the complexity of the organoids (e.g., addition of microglia and vasculature). We envision that the adoption of organoid systems will be beneficial to researchers studying mechanisms of brain infection by protozoan parasites.”* Although it is important to showcase the limitations of the model, the second part of the sentence has somehow a negative connotation on the validity of the current results. In this paper, the authors validate the use of organoids systems to study *T. brucei *infection and the authors should highlight that they already represent an alternative to animal models.
- Research highlights (3Rs benefit(s)) and results page 3: Which methodology did you use to measure the reduction in animal models that could be used to study trypanosomiasis?
- There seems to be a reference missing in: *“For instance, there are intrinsic differences in the immune response between hosts (e.g., mouse vs. human) that pose limitations for translational science.”*
- Results Page 7: “ *We also detected significant expression of the interleukin-17 receptor subunit A* *and D (IL17RA and IL17RD, respectively), interleukin-10 receptor subunit a (IL10RA), and the Interferon gamma receptor 1 (IFNGR1) (Table 1 and Table S2 in Underlying data),22, indicating that these organoids are primed to sense and respond to IL-17, IL-10, and IFNγ signalling upon exposure to T. brucei gambiense” -* I would recommend to rephrase to something similar to “these organoids are prime to sense and response to *T. brucei gambiense *by activating IL-17, IL-10 and IFNg pathways.”

Is the rationale for developing the new method (or application) clearly explained?

Yes

Are the 3Rs implications of the work described accurately?

Yes

Is the description of the method technically sound?

Yes

Are the conclusions about the method and its performance adequately supported by the findings presented in the article?

Yes

If any results are presented, are all the source data underlying the results available to ensure full reproducibility?

Yes

Are a suitable application and appropriate end-users identified?

Yes

Are sufficient details provided to allow replication of the method development and its use by others?

Partly

Reviewer Expertise:

3D brain bioengineered models, host-parasite interactions, malaria, vascular engineering

I confirm that I have read this submission and believe that I have an appropriate level of expertise to confirm that it is of an acceptable scientific standard, however I have significant reservations, as outlined above.

# Response

**Published:** 2023-07-22

**Authors:** Juan Quintana

**Affiliations:** University of Glasgow, UK

In this manuscript, Chandrasegaran *et al.*, use for the first-time *in vitro *brain organoids to understand *Trypanosoma brucei gambiense *pathogenesis. This is an important topic, as brain organoids have already become as a valid strategy to study disease mechanisms. Although the team led by Juan Quintana is already a leader in animal models of *T. brucei,* I would like to praise their efforts on using alternative disease modelling tools that could reduce the number of research animals and overcome differences between mouse and humans. The authors show differential gene expression after brain organoid incubation with *T, brucei gambiense *after a 24h and 72h incubation. The manuscript is timely, and overall, scientifically sound and well written. I support the article indexing after the introduction of some modifications on the text.

**We sincerely thank this reviewer for their positive assessment of our work. We are always motivated to explore novel and more ethical ways to conduct our research and are indeed excited by the possibility of implementing organoids and “organ-on-a-chip” approaches to study brain responses to infection.**

**Major**

- Although the methods section is very clear, I couldn’t find any reference on how long the brain organoids were grown. This is important because brain organoids are grown for long periods of time (up to a year) and depending on the maturation time, they acquire different brain developmental stages. I would suggest that the authors include this information in the methods section, as well as on the results or discussion section, as it is important for interpretation of the results.

**Authors: We apologise for this issue. The cortical brain organoids used in this study were ~2 months old, to ensure a minimum level of maturation, as reported in references 2 and 4. We have included this in the methods section of the revised manuscript.**

- How does the 24h and 72h time point or the parasite concentration used compare with the life cycle of *T. brucei gambiense* in the brain? This information could be useful for an audience not familiar with *T. brucei*.

**Authors: This is a great question, but a challenging one to address. Gambiense HAT is  typically mild, lasting for years before the clinical symptoms associated with the second stage of the disease (e.g., sleep disturbances) become patent. Unfortunately, there is no clinical data indicating how many parasites there are in the brain at any given points during gambiense HAT, but CSF from second stage gambiense HAT patients can report anywhere tens to hundred parasites per ml of CSF ( https://www.thelancet.com/journals/ebiom/article/PIIS2352-3964(22)00558-8/fulltext for reference). In our own experience infecting mice with *T. b. gambiense*, we typically observed around 10 ^2^-10 ^4 ^parasites/gram of brain tissue (unpublished data), but we are unsure at this stage how comparable this is to the *in vitro *system we set out to develop here. We will focus on these aspects in future work. **

- The authors couldn’t find any brain damage markers on Fig 2C after staining with two different markers. However, other non-used markers could be affected. Although the sentence in the result section mildly implies that (" *Importantly, we did not detect significant morphological or histological changes in the organoids exposed to the parasites based on H&E staining and MAP 2 staining (Figure 1C), suggesting that T. brucei gambiense does not elicit tissue damage over a 72 hour culture period.*”), I would suggest to rephrase to make it more obvious. Furthermore, did the author quantify for differences in expression on MAP2, or is the analysis mostly qualitative?

**Authors: We thank this reviewer for this useful suggestion. We have rephrased the text to highlight that we are basing our observations of a limited number of morphological markers (H&E and MAP2), as follow: *Importantly, we did not detect significant morphological or histological changes in the organoids exposed to the parasites based on the morphological aspects observed upon H&E staining and MAP2 staining ( Figure 1C), suggesting that T. brucei gambiense does not seem to elicit tissue damage over a 72 hour culture period, at least not with the markers used in this study*.**

**It is important to note that we originally intended to keep some of these organoids to run western blotting for quantification of markers of interest (e.g., MAP2) but were unable to do so due to limited availability. We plan to dissect this aspect in more detail as part of future work.**

- The increased expression of endothelial markers is intriguing, taking into account that brain organoids present minimal presence of endothelial cells. This is important because vascularization of brain organoids is one of the main challenges in the bioengineering field, and the presence of vasculature in these models could be used to study the molecular mechanisms of *T. brucei* crossing through the brain microvasculature. Did the authors checked for the presence of blood vessels by immunostaining. In a similar topic, I would like to clarify that EPCAM is not an endothelial marker, so I would recommend to delete any reference to EPCAM in the figures/tables, results and discussion section.

**Authors: We agree with this reviewer. We were also surprised to see an upregulation of genes typically found in the endothelial compartment as these organoids do not typically contain vasculature, as this reviewer states. It is important to note that we failed to detect positive staining for VWF and CDH5 by immunohistochemistry, but there might be technical limitations to consider (e.g., antibody compatibility, detection level, etc) before we can confidently support or exclude the presence of a vasculature-like cell population in our dataset in response to *T. b. gambiense* exposure/co-culture. Although intriguing and exciting, we decided to err on the side of caution and limit our interpretations to present the transcriptional features, without concluding that these organoids in fact contain vasculature. Also, as suggested by this reviewer, we have removed references to *Epcam* from the text/table.**

**Minor**

- Abstract *“Future work is required to increase the complexity of the organoids (e.g., addition of microglia and vasculature). We envision that the adoption of organoid systems will be beneficial to researchers studying mechanisms of brain infection by protozoan parasites.”* Although it is important to showcase the limitations of the model, the second part of the sentence has somehow a negative connotation on the validity of the current results. In this paper, the authors validate the use of organoids systems to study *T. brucei *infection and the authors should highlight that they already represent an alternative to animal models.

**Authors: We agree with this reviewer. As suggested, we have amended the abstract accordingly as follow: *Although our data support the use of brain organoids to explore and model host-pathogen interactions in the context of T. brucei infection as an alternative to in vivo models, future work is required to increase the complexity of the organoids ( e.g., addition of microglia and vasculature). We envision that the adoption of organoid systems is beneficial to researchers studying mechanisms of brain infection by protozoan parasites*.**

- Research highlights (3Rs benefit(s)) and results page 3: Which methodology did you use to measure the reduction in animal models that could be used to study trypanosomiasis?

**Authors: The figures presented in the current version of the manuscript were based on a prediction assuming full adoption of this method, and the number of animals that undergo similar procedures (e.g., brain responses to infection) over the past 5 years of our current UK Home Office license. In this case, we determined that on a typical year of our license, 47/100 animals undergo moderate-to-severe procedures involving evaluating brain responses to infection that could be fully replaced with a suitable *in vitro* brain organoids system. To generate those infections, we also typically require donor mice from which we obtain infective trypanosomes, which we estimated to be reduced by about 1/5 with the organoids system in place. **

- There seems to be a reference missing in: *“For instance, there are intrinsic differences in the immune response between hosts (e.g., mouse vs. human) that pose limitations for translational science.”*

**Authors: We have removed this sentence from the manuscript. **

- Results Page 7: “ *We also detected significant expression of the interleukin-17 receptor subunit A and D (IL17RA and IL17RD, respectively), interleukin-10 receptor subunit a (IL10RA), and the Interferon gamma receptor 1 (IFNGR1) (Table 1 and Table S2 in Underlying data),22, indicating that these organoids are primed to sense and respond to IL-17, IL-10, and IFNγ signalling upon exposure to T. brucei gambiense” -* I would recommend to rephrase to something similar to “these organoids are prime to sense and response to *T. brucei gambiense *by activating IL-17, IL-10 and IFNg pathways.”

**Authors: We have rephrased the manuscript as suggested as follow: *indicating that these organoids are primed to sense and respond to T. brucei gambiense by activating IL-17, IL-10, and IFNγ signalling pathways***

# Peer review: Reviewer response for version 1

**DOI:** 10.5256/f1000research.144355.r170892

**Published:** 2023-05-03

**Authors:** David Smith

**Affiliations:** 1 Moredun Research Institute, Penicuik, Scotland, UK

This manuscript from Chandrasegaran et al., reports the application of iPSC-derived cerebral organoids as a model for neurological *T. b. gambiense* infection. Organoids are increasingly powerful *in vitro* models that better represent *in vivo* tissues compared to simpler cell culture systems. This is allowing researchers to address research questions at a level of precision and detail that is difficult to achieve *in vivo*. It also greatly reduces reliance on animals to experimentally address specific research questions. Overall, the methods in this manuscript are very clear (some minor points to be addressed stated below) which, importantly, provides an accessible protocol for other researchers to follow for the cultivation of iPSC-derived cerebral organoids (for which the authors are commended). In the results, the authors demonstrate infection of cerebral organoids by *T. b. gambiense* parasites and the differential gene expression determined between uninfected organoids and organoids infected with *T. b. gambiense* for 24 or 72 hours. A large number of genes are differentially up and down regulated and the author’s inclusion of GO analysis is helpful to provide broader context to these transcriptomics differences. This report is overall very clear (with some minor points to address below) and will provide a valuable addition to the literature. There is minimal modification necessary before this manuscript is suitable for indexing.

1. How long were the cerebral organoids cultivated for before challenge with parasites? Were the consistently matured to the same started point? What is the timescale for “maturing” the cerebral organoids?
2. Point 1 under “ *Immunohistochemistry”*(methods) should read “storage” instead of “maintenance”.
3. The results section indicates 3 experimental replicates were used in the RNA-seq analysis for each test group. Please make this clear in the associated methods section.
4. Perhaps there is not sufficient read depth, but if it is possible it would be interesting to map the parasite RNA-seq reads from the 24h and 72h group to determine any differences, potentially informing on how the parasite is adapting to co-culture and infection in the cerebral organoids and whether this is similar to what would be expected/observed in vivo. If this is possible, it would have been useful to have a test group of parasites in the RNA-seq analysis representative of parasites immediately prior to organoid challenge (to better assess how the parasite itself is responding to co-culture with the organoids) – this is just a point for future consideration for the authors. It would be useful in future experiments to see how long a chronic infection can persist in the organoids (i.e. how far beyond 72 hours post-challenge can the cultures be maintained?).
5. How did the authors determine the predicted reduction in animal usage by replacement with organoids.
6. Figure legend 1C should say (left) and (right) instead of (top) and (bottom)? Also use arrows in panel C to indicate parasites for non-specialists.
7. Did the authors quantify parasite burden in the cerebral organoids at 24 and 72 hours post-challenge, relative to the 10 ^5^ parasites used to challenge organoids at the start of the experiment.

Is the rationale for developing the new method (or application) clearly explained?

Yes

Are the 3Rs implications of the work described accurately?

Yes

Is the description of the method technically sound?

Yes

Are the conclusions about the method and its performance adequately supported by the findings presented in the article?

Yes

If any results are presented, are all the source data underlying the results available to ensure full reproducibility?

Yes

Are a suitable application and appropriate end-users identified?

Yes

Are sufficient details provided to allow replication of the method development and its use by others?

Partly

Reviewer Expertise:

Organoids, parasitology, host:parasite interactions, protozoa, helminths

I confirm that I have read this submission and believe that I have an appropriate level of expertise to confirm that it is of an acceptable scientific standard, however I have significant reservations, as outlined above.

# Response

**Published:** 2023-07-22

**Authors:** Juan Quintana

**Affiliations:** University of Glasgow, UK

This manuscript from Chandrasegaran et al., reports the application of iPSC-derived cerebral organoids as a model for neurological *T. b. gambiense* infection. Organoids are increasingly powerful *in vitro*models that better represent *in vivo* tissues compared to simpler cell culture systems. This is allowing researchers to address research questions at a level of precision and detail that is difficult to achieve *in vivo*. It also greatly reduces reliance on animals to experimentally address specific research questions. Overall, the methods in this manuscript are very clear (some minor points to be addressed stated below) which, importantly, provides an accessible protocol for other researchers to follow for the cultivation of iPSC-derived cerebral organoids (for which the authors are commended). In the results, the authors demonstrate infection of cerebral organoids by *T. b. gambiense* parasites and the differential gene expression determined between uninfected organoids and organoids infected with *T. b. gambiense* for 24 or 72 hours. A large number of genes are differentially up and down regulated and the author’s inclusion of GO analysis is helpful to provide broader context to these transcriptomics differences. This report is overall very clear (with some minor points to address below) and will provide a valuable addition to the literature. There is minimal modification necessary before this manuscript is suitable for indexing.

**Authors: We sincerely thank this reviewer for taking the time to provide feedback on our methods article. We hope the comments below clarify their questions.**

1. How long were the cerebral organoids cultivated for before challenge with parasites? Were the consistently matured to the same started point? What is the timescale for “maturing” the cerebral organoids?

**Authors:** **The cortical brain organoids used in this study were ~2 months old, to ensure a minimum level of maturation, as reported in references 2 and 4.**

1. Point 1 under “ *Immunohistochemistry”*(methods) should read “storage” instead of “maintenance”.

**Authors:** **We have amended the text accordingly.**

1. The results section indicates 3 experimental replicates were used in the RNA-seq analysis for each test group. Please make this clear in the associated methods section.

**Authors:** **We have clarified this in the methods section, in step 2 under “Bulk RNA sequencing and data analysis”.**

1. Perhaps there is not sufficient read depth, but if it is possible it would be interesting to map the parasite RNA-seq reads from the 24h and 72h group to determine any differences, potentially informing on how the parasite is adapting to co-culture and infection in the cerebral organoids and whether this is similar to what would be expected/observed in vivo. If this is possible, it would have been useful to have a test group of parasites in the RNA-seq analysis representative of parasites immediately prior to organoid challenge (to better assess how the parasite itself is responding to co-culture with the organoids) – this is just a point for future consideration for the authors. It would be useful in future experiments to see how long a chronic infection can persist in the organoids (i.e. how far beyond 72 hours post-challenge can the cultures be maintained?).

**Authors: Indeed, we agree with this reviewer, and this is something we plan to consider in future experiments. We had financial constraints that limited our ability to explore how the parasites responded to the co-culture system. It is important to note that in our hands, we did not see parasites penetrating the organoids *per se*, and so we decided to wash the organoids with 1X PBS to remove as many parasites as possible. This has now been clarified in the methods section, in step 3 under “Trypanosoma brucei gambiense – human brain organoids co-culture system”.**

1. How did the authors determine the predicted reduction in animal usage by replacement with organoids.

**Authors:** **The figures presented in the current version of the manuscript were based on a prediction assuming full adoption of this method, and the number of animals that undergo similar procedures (e.g., brain responses to infection) over the past 5 years of our current Home Office license. In this case, we determined that on a typical year of our license, 47/100 animals undergo moderate-to-severe procedures involving evaluating brain responses to infection that could be fully replaced with a suitable *in vitro* brain organoids system. To generate those infections, we also typically require donor mice from which we obtain infective trypanosomes, which we estimated to be reduced by about 1/5 with the organoids system in place.**

1. Figure legend 1C should say (left) and (right) instead of (top) and (bottom)? Also use arrows in panel C to indicate parasites for non-specialists.

**Authors:** **We have amended the figure legend as suggested. Unfortunately, we did not detect parasites inside the organoids by any means, and so the images in panel 1C are showing the integrity of the organoids post-culture compared to untreated organoids, and the staining for neurons using MAP2.**

1. Did the authors quantify parasite burden in the cerebral organoids at 24 and 72 hours post-challenge, relative to the 10 ^5^ parasites used to challenge organoids at the start of the experiment.

**Authors: In short, we did not. We had originally intended to inoculate the parasites inside the organoids using microinjections, but this was not feasible for various reasons (e.g., access to adequate microinjection platforms, size of the organoids, etc). Thus, we decided to co-culture the parasites with the organoids instead. Before doing so, we ensured that the parasite remained viable under the culture conditions (50:50 organoids:HMI9 media) using a growth curve approach, as shown in figure 1B.**
